# A Cross-sectional study design of risk factors related to antenatal care service use among pregnant women in Sinana district, Bale zone, Ethiopia

**DOI:** 10.4314/ahs.v23i4.51

**Published:** 2023-12

**Authors:** Meskerem Abebe, Alemayehu Legesse, Getu Dida, Habtamu Tedila

**Affiliations:** 1 Department of Statistics, Madda Walabu University, Bale Robe, Ethiopia; 2 Department of Biology, Madda Walabu University, Bale Robe, Ethiopia

**Keywords:** Antenatal care, Monte Carlo estimation method, Bayesian multilevel logistic regression

## Abstract

**Background:**

Maternal health refers to the well-being of women through pregnancy, childbirth and the postpartum period. Antenatal care refers to the care that is given to a pregnant woman from the time that conception is confirmed until the beginning of labour. Ethiopia was known in concert of the world's nation with primary maternal mortality proportions. The major goal of this study was to survey risk factors related with antenatal care service use among pregnant ladies at regenerative age.

**Methods:**

Data were obtained from primary sources. To dissect the information, descriptive and Bayesian multilevel binary logistic regression of random coefficient model was utilized. The convergence of parameters was assessed by Monte carol Markova Chain utilizing Stata 16 and MLwiN 2.31 programming.

**Results:**

The descriptive result showed that out of the whole 636 pregnant women considered around 60.5%were obtained antenatal care benefits. The odds ratio of pregnant women living in the rural areas being receiving antenatal care services was 0.206(OR = 0.206, P ≤ 0.05). This indicates that the use of prenatal care by rural mothers has decreased by 79.4% compared to urban mothers. Similarly, the Odds ratio for Pregnant women with medium and rich wealth index are 1.571(OR = 1.571, P ≤ 0.05) and 1.90(OR = 1.90, P ≤ 0.05) respectively. This means that pregnant women who are with medium and rich wealth index level had 57.1% and 90% increased odds compared to those pregnant women with poor wealth index consecutively. Varieties between the kebeles in terms of antenatal care benefit utilize were lessening by 0.9 % in random coefficient model.

**Conclusion:**

Generally, the study showed that there was high variation among pregnant women not to utilize antenatal care benefits and the likelihoods of prenatal use were found to increase with the husband occupation, wealth index, age category 25-34, and husband education level.

## Background of the Study

Maternal morbidity and mortality remained major concerns in many countries. Between 1990 and 2015, a total of 10.7 million women died as a result of maternal causes worldwide, with sub-Saharan Africa accounting for 66% of maternal deaths. More than 80% of maternal deaths can be avoided if pregnant women have access to essential maternity care, such as antenatal care 3, 6.

Antenatal care (ANC) can be expressed as one of the center services conveyed by experienced health-care suppliers to move forward pregnant women and youthful girl's wellbeing conditions for both mother and child amid pregnancy 10, 14. Maternal health depicts in terms of well-being of women through gestation, parturition and therefore the postpartum period. Women in advanced countries have access to elementary health care whereas those survive in developing countries associated with suffering, health problem and even death. It's vital for the societies, families and Country as its preponderating result on the welfare of women 5.

## Methods and materials

The target population of this study was all pregnant women at reproductive age, 15-49, living in Sinana district. The data source implemented in the study was primary. Thus, a well-structured questionnaire was prepared to get valuable information. The sampling method applied in this study was the cluster-sampling technique. First, the target population of the study was divided into different groups called clusters, it is assumed that there is heterogeneity in responses of members within the clusters. Thus, a randomly selected sample cluster can represent the whole target population.

### Variables included in the study

The response variable of interest of this study was antenatal care use service. For the purpose of the study, this response variable can be dichotomizing as 1, if the pregnant women use ANC service (those pregnant women who were receiving Antenatal care service during the study time), and 0, otherwise (if not used). This study has tried to include the most important expected determinants of ANC service among pregnant women from various literature reviews. Those were religion, Age, employment status of women, employment status of the husband, birth order, husband's education status, attitudes of husband, marital status, mother's educational status, women exposure to media, years living with husband, and wealth index.

### Bayesian multilevel binary logistic regression model

Data with a multilevel or hierarchical structure occur frequently in a wide range of research disciplines. For instance, in a study of reproductive age women Antenatal care service, women are clustered within kebele, which in turn may be clustered within district 2. Multilevel models are also called hierarchical, for two different reasons: first, from the structure of the data (for example, women clustered within kebele); and second, from the model itself, which has its own hierarchy, with the parameters of the within-kebele regressions at the bottom, controlled by the hyper parameters of the upper-level model^1^.

In conventional multilevel data, each level one unit (e.g., women) is nested in one and only one level two unit (e.g., kebele). The level two units may subsequently be nested in one and only one level three units (e.g., woreda). Further levels of clustering or nesting are possible. The most common multilevel model is a two-level hierarchic nested modeling with many level-1 units within a smaller number of level-2 units. A multilevel structure can be cast, with great advantage, to incorporate a range of circumstances where one may anticipate clustering 14.

In this study, we restrict our attention to multilevel data with two levels. The outcome or response variable is measured at the lowest level of the hierarchy on the level one unit, whereas explanatory or predictor variables can be measured on units at any of the levels of the hierarchy 2. Hierarchical models can account for lack of independence across levels of nested data. These models can be used to analyse nested sources of variability in hierarchical data, taking in to account the variability associated with each level of the hierarchy. These models have also been referred to as multilevel models, mixed models, random coefficient models, and covariance component models 4

In this study, two level models are employed that accounts women and kebele level effects. There are *n_j_* individuals within each level two unit in the *J^th^* kebele. The model can be written as:


$$\text{log}\left(\frac{P_{ij}}{1-P_{ij}}\right)=\beta_{0}+U_{0}+(\beta_{1}+U_{1})x_{1}+\ldots +(\beta_{k}+U_{k})X_{k}\ldots \ldots \ldots \ldots \ldots \ldots \ldots \ldots \ldots 1$$

Where:

$U∼N(0,\sigma_{u}^{2})$, is random effect at level-2

*β*_0_, *β*_1_, …, *β*_*k*_ are coefficient

Conditional on *U_j_*, then *y_ij_* is assumed to be independent. Then level one and level two models can be expressed as follows respectively.


$$\text{log}(P_{ij})=\text{log}\left(\frac{P_{ij}}{1-P_{ij}}\right)=\beta_{0j}+\beta_{i}x_{ij}\ldots \ldots \ldots \ldots \ldots \ldots \ldots 2$$

$$\beta_{0j}=\beta_{0}+U_{j}\ldots \ldots \ldots \ldots \ldots \ldots \ldots \ldots \ldots \ldots \ldots \ldots \ldots \ldots \ldots \ldots 3$$

Let the success probability in kebele j be denoted by *P_j_*. The dichotomous outcome variable for the individual i in group j, *y_ij_*; which is either 0 or 1 can be expressed as sum of the probability in group j, *p_j_* (the average proportion of j levels in group j, (*E*(*y_ij_*) = *p_j_*) plus some individual dependent residual *ε_ij_*, that is, *y_ij_* = *p_j_* + *ε_ij_* the residual term is assumed to have mean zero and variance, var(*ε_ij_*) = *p_j_* (1 − *p_j_*)

### Intraclass correlation coefficient

The intra-class correlation coefficient (ICC) measures the proportion of variance in the response variable that is explained by the grouping structure. i.e., the authors were used it to examine the proportion of ANC service use variation that occurs across kebeles (i.e., level-2 units). Then, a two-level random intercept multilevel logistic regression model was employed to get it.


$$ICC=\frac{\sigma_{0}^{2}}{\sigma_{0}^{2}+\sigma_{e}^{2}}$$

Where: $\sigma_{e}^{2}$ individual women variation at Level 1.

### Bayesian multilevel binary logistic regression random coefficient model

In the random coefficient model, the authors assumed that the effects of explanatory variables vary across higher-level units (kebele). In this study, for example, the Authors might expect that kebeles are differing in their effect on ANC use of pregnant women according to involved explanatory variables. This can be done considering different slopes for the relation between logit of outcome and explanatory variable by introducing intercept residual and slope residual at the higher-level unit (kebele). This model is based on linear models for the log odds that include random effects for groups or other higher levels.


$$\text{log}it(P_{ij})=\text{log}\left(\frac{P_{ij}}{1-P_{ij}}\right)=\beta_{0j}+\sum_{k=1}^{p}\beta_{k}X_{kij}+\sum_{r=1}^{q}U_{rj}X_{qij}\ldots \ldots \ldots \ldots \ldots \ldots \ldots \ldots 4$$

Where: *k* = 1, 2 … *p*, is the number of explanatory variables at level-1 (e.g., women) whereas *r* = 1, 2, …, *q* is number of explanatory variables measured at level-2(kebele)

Then *β_σj_* can be expressed as:


$$\beta_{0j}=\beta_{0}+U_{0j}\ldots \ldots \ldots \ldots \ldots \ldots \ldots \ldots \ldots 5$$

As a result, equation (4) can be written as:


$$\text{log}it(P_{ij})=\text{log}\left(\frac{P_{ij}}{1-P_{ij}}\right)=\beta_{0}+\sum_{k=1}^{p}\beta_{k}X_{kij}+U_{0j}+\sum_{r=1}^{q}U_{rj}X_{qij}\ldots \ldots \ldots \ldots \ldots \ldots 6$$

Where the random effect *U*_0*j*_ *and U_ij_* are identically and independent distributed as with both mean zero and respective variance, $\sigma_{0}^{2}$ and $\sigma_{r}^{2}$ respectively. In this study the authors assumed that the effects of these random parts are independent between higher levels (in this study, kebele) but may not within kebele.

Bayesian approach develops a full probability distribution over all data and population parameters, fitting the model to observed data, then reasoning about either the fitted parameters or about new data taking into account the uncertainty in the fitted parameters. The main difference between classical multilevel logistic regression and Bayesian approaches are that the former treats the unknown parameters of interest as fixed constants for a fixed effect and treats as random for random effect without assuming any probability distribution, while the later considers every unknown parameter as random variable, which means it assigns probability distribution to the unknown parameters of interest.

One of the key aspects in Bayesian inference is identifying the probability distribution that represents the prior information related with the population parameters. A prior distribution that is used based on the availability of information about the likely values of the unknown parameters before the data is collected considered as informative prior. It obtained from expert knowledge, historical belief and combination of both. However, in some cases we might prefer not to use this information obtained about the coefficient value and let see what the data themselves provide as inferences. This leads to non-informative priors which provide little information relative to the experiment. In this study the Authors employed normal distribution prior for the fixed effect (*β*) and Inverse gamma Prior for random effect (*σ*^2^ *µ*).

### Posterior distribution

The posterior density stands for your beliefs about the parameter of interest given your prior beliefs and the beliefs encompassed in the likelihood. In many applications the posterior is the ending of the empirical analysis. Reporting the results of the empirical analysis imply displaying the posterior distribution, to which the model and data have led, which can be done in several ways. In Bayesian approach, inferences made based on the sampling from posterior distribution until the convergence is achieved. The main delinquent in Bayesian setting is posterior distribution may not belong to exponential family distribution. Due to this reason, employing MCMC Simulation methods are mandatory. Simulation is a general computational method in Bayesian inference to obtain the posterior distribution of the parameters. This method is based on drawing values of parameters β from approximate distributions.

### Parameter estimation

The maximum likelihood (ML) method is a general estimation procedure, which produces estimates for the population parameters that maximize the probability of observing the data that are actually observed. Assuming that the conditional distributions of *Y_ij_* given the random effect, *U_ij_* are independent of each other, the conditional density of *Y_ij_* is given by *p_ij_*:


$$Y_{ij}|U_{j}=\left(\frac{y_{ij}}{u_{ij}}\right)∼bernoulli\ldots \ldots \ldots \ldots \ldots \ldots \ldots 7$$

For two-level logistic Bernoulli response model, where random effects are assumed to be multivariate normal and independent across units, the marginal likelihood function is given by:


$$l(\beta ,\Omega )=\prod_{i}f\prod_{i}(\pi_{ij}{)}^{y_{ij}}(1-\pi_{ij}{)}^{1-y_{ij}}\ldots \ldots \ldots \ldots \ldots \ldots \ldots 8$$

Where: Ω variance covariance matrix.


$$\pi_{ij}=\lfloor 1+\text{exp}(-x_{ij}\beta_{j})\rfloor \ldots \ldots \ldots \ldots \ldots \ldots \ldots 9$$

$$\beta_{j}=\beta +U_{j}\ldots \ldots \ldots \ldots \ldots \ldots \ldots \ldots \ldots \ldots \ldots 10$$

*f*(*U*_*ij*_, *Ω*), typically assumed to be the multivariate normal density and can be written in the form $\int p(U_{j})f(U_{j})du_{j}$.

### Statistical analysis and quality checking methods

The Primary data obtained through questionnaire were checked for completeness and consistency by the principal investigators. Data entry and analyses were carried out using SPSS version 20 and MLwiN. Descriptive statistical methods were used to summarize the socio-demographic characteristics of the study participants. Cross-tabulation was used to identify the relationship between the variables. Bayesian Multilevel binary logistic regression random coefficient model was used to identify risk factors of Antenatal care service use of pregnant women in Sinana district using forward stepwise procedure. Variables showing significant association (P < 0.25) in the uni-variate analyses were included in the multiple variables analysis of random coefficient Model. Odds ratio (OR) and corresponding 95% confidence interval (95%CI) were estimated for Significant risk factors included in the multiple variable models.

## Results

### Descriptive statistics results

According to descriptive results displayed in table 1, A total of 636 pregnant women were considered in this study. Out of these pregnant women involved in the analysis, 60.5% were receiving Antenatal care service while 39.5% were not receiving the service at the time of data collection. Of the total sample, 47.6% of the married pregnant women use Antenatal care service regularly, 5.9% Divorced pregnant women use ANC service while 5.8% do not, 6% Widowed those use the service and 2.4% do not. With regard to the kebele of the respondents, the highest proportion of women that were not receiving ANC service was observed in Obora, 24(3.8%) followed by Hissu, 19(3%). In the case of Alemgena town, Hassenbarera, Hamida, and Shalo the proportion of women that were not receiving ANC service was (0.3%), (1.9%), (2.4%), and (1.9%) respectively. The majority of the participants, (20.1%) primary, and the rest are Secondary (15.3%) or Diploma and above education level (1.3%) were not Receiving ANC service during the study time. The proportion of ANC service differs by type of occupation of pregnant women. Accordingly, about more than half of the pregnant women (64.3 percent) Household workers unlike the relatively smaller number of pregnant women at reproductive age (7.1 percent) who participate in informal market.

**Table 1:** Descriptive Analysis results showing relation between utilization of antenatal care Services and socio-demographic and economic variables

Variable	Category	Use of antenatal care service			
		Yes	No	Total	%
**Age**	15-19	14	13	27	4.2
	20-24	46	30	76	11.9
	25-29	61	35	96	15.1
	30-34	79	46	125	19.7
	35-39	84	58	142	22.3
	40-44	71	41	112	17.6
	45-49	30	28	58	9.1
**Marital status**	Single	7	24	28	4.9
	Married	303	175	478	75.2
	Divorced	38	37	75	11.8
	Widowed	37	15	52	8.2
**Women educational level**	No education	12	18	30	4.7
	Primary	125	128	253	39.8
	Secondary	169	97	266	41.8
	Diploma and above	79	8	87	13.7
**Religion**	Orthodox	128	69	197	31.0
	Muslim	150	129	279	43.9
	Protestant	70	27	97	15.3
	Other	37	26	63	9.9
**Husband educational level**	No schooling	8	12	20	3.1
	Primary	93	100	193	30.3
	Secondary	192	129	321	50.5
	Diploma and above	92	10	102	16
**Occupation of women**	House hold worker	236	173	409	64.3
	Merchant	51	44	95	14.9
	Informal market	19	26	45	7.1
	Employed	79	8	87	13.7
**Number of Children they have**	No children	40	21	61	9.6
	1-3	149	74	213	33.5
	4-6	139	74	223	35.1
	More than 6	57	82	139	21.9
**Husband's attitude towards maternal health care services**	Positive	333	84	417	65.6
	Negative	52	167	219	34.4
**Number of weeks visited by the community health institution**	0-8	21	194	215	33.8
	9-16	110	13	123	19.3
	17-24	83	11	94	14.8
	25-32	99	18	117	18.4
	33-40	72	15	87	13.7
**Exposed by mass media**	Yes	298	71	369	58.0
	No	87	180	267	42.0
	Usually,	251	0	251	39.5
	Sometimes	134	0	134	21.1
	Never	0	251	251	39.5
**Advice before to start the service care**	Yes	371	19	390	61.3
	No	20	226	246	38.7
**From whom you get advice**	Health professional	173	6	179	28.1
	Husband	34	1	35	5.5
	Friends	73	12	86	13.5
	Family	48	7	56	8.8
	Mass media	28	11	39	6.1
	From no one	29	214	241	37.9
**Get education on maternal health care service**	Yes	343	38	381	59.9
	No	42	213	255	40.1
**Transportation to the health stations**	On foot	218	36	254	39.9
	Vehicles	24	1	25	3.9
	Cart	143	12	155	24.5
	I don't use any one	0	202	202	31.6
**Comfort ability of road to use antenatal care service**	Yes	98	13	111	17.5
	No	287	238	525	82.5
**Awareness on the difficulty of pregnancy**	Yes	351	82	433	68.1
	No	34	169	203	31.9
**Occupation of husband**	Farmer	207	167	374	58.8
	Merchant	62	41	103	16.2
	Employed	76	9	85	13.4
	Informal market	21	16	37	5.8
	Other	19	18	37	5.8
**Income of respondents per month**	Low	92	61	153	24.1
	Medium	247	154	401	63.1
	High	46	36	82	12.9
**Perception regarding quality of health institution in sinana woreda**	Very bad	22	11	33	5.2
	Bad	46	15	61	9.6
	Good	251	50	301	47.3
	Very good	66	16	82	12.9
	I do no	0	159	159	25.0
	W/Arjo	22	13	54	8.5
**Keble's of respondents**	W/Web	13	11	24	3.8
	Hisu	28	19	47	7.4
	Obora	30	24	54	8.5
	Alemgena Town	7	2	9	1.4
	Hassenbarera	23	12	35	5.5
	Hamida	11	15	26	4.1
	Shallo	20	12	32	5.0
	Selka Rural	13	9	22	3.5
	Selka Town	20	10	30	4.7
	Besmenna	9	5	14	2.2
	Gemora	13	6	19	3.0
	IlluSenbitu	28	17	45	7.1
	Alage	16	13	29	4.6
	Shawwade	13	7	20	3.1
	KabiraTemo	21	13	34	5.2
	WaltaiBarisa	12	5	17	2.7
	Hawusho	20	11	31	4.9
	S/Robe	18	10	28	4.4
	H/Boka	17	14	31	4.9
	Basaso	14	8	22	3.5
	K/ Shekmara	18	14	32	5.0

**Note:** H/Boka= Hora Boka, K/Shekmara = Kaso Shekmara, S/Robe= Surrounding Robe, W/Arjo= Weltai Arjo, W/web = Weltai Web

Likewise, descriptive results displayed in table 1 show, Antenatal care use services among pregnant women were varied by the number of children they have. The highest proportion of ANC service use was observed from pregnant women with 1-3 children (23.4%) followed by pregnant women with 4-6 children (21.9%). The lowest proportion of ANC service use was recorded from mothers with no children (6.3%) followed by pregnant women with more than six children (9 percent). The results displayed in Table 1 also show that the proportion of ANC service use of pregnant women varied with respect to their income status. The highest Proportion of ANC service use was observed among Mothers belonging to medium-income levels (38.8 percent) as opposed to the lowest Proportion of ANC service use which was recorded from mothers in high-income status (7.2 percent). According to descriptive results displayed in Table 1, the proportion of ANC service use of pregnant women also differs with their age groups. For instance, the highest proportion of ANC use was observed among pregnant women under 35-39 years of age (13.2%), and the lowest was in the age interval 15-19 years (2.2%).Based on road comfortability, Out of the integrated sample, only about 15.4% of pregnant women got a comfortable road for transportation to use antenatal care service in sinana woreda whereas about 45.1 percent of pregnant women did not get the suitable road to follow service during data collection time in Sinana district. A Similar interpretation for the rest variables (See Table 1).

### Bayesian multilevel logistic regression of random coefficient model

Bayesian multilevel logistic analysis procedure was used to make inference about the parameters of a multilevel logistic model. It permits incorporation of prior beliefs and the combination of such beliefs with statistical data. Due to this it well suited for representing the uncertainties in the value of explanatory variables. In this model levels one predictors have different effect across the region (that means the effect of the coefficient of predictor variables at lower level to vary from kebele to kebele). This can be done by allowing random coefficients at level-1 covariates of the model.

The results of the Bayesian multilevel random coeffcient model displayed in table 2 indicate that place of residence, wife beat, birth order, religion, husband occupation, Wealth index, and husband education have a statistically significant effect on the usage of ANC services of pregnant women in Bale Zone Sinana district. Thus, we can interpret the results as follow. Odds ratio interpretation in ordinary logistic and multilevel logistic regression is nearly similar except random parts (further information) that added into multilevel logistic regression.

**Table 2:** Results of random coefficient Bayesian multilevel logistic regression model

Fixed effect	$\hat{\beta }$	Exp ($\hat{\beta }$)	S. E	Z-value	P-value
Constant	1.696	5.452	0.212	8.00	P ≤ 0.05
Place of residence					
Urban (ref)					
Rural	-1.578	0.206	0.171	9.228	P ≤ 0.05
**Beat**					
No(ref)					
Yes	-0.146	0.864	0.067	2.179	P ≤ 0.05
**Age**					
15-24(ref)					
25-34	0.124	1.132	0.105	1.181	P ≤ 0.05
35-49	-0.083	0.92	0.133	0.624	P ≤ 0.05
**Birth order**					
1-4(Ref)					
5-8	-0.23	0.795	0.093	2.473	P ≤ 0.05
>8	-0.33	0.719	0.159	2.075	P ≤ 0.05
**Religious**					
Orthodox(ref)					
Catholic	-1.094	0.335	0.451	2.426	P ≤ 0.05
Protestant	-0.707	0.493	0.147	4.809	P ≤ 0.05
Muslim	-0.473	0.623	0.134	3.529	P ≤ 0.05
Other	-1.375	0.252	0.313	4.393	P ≤ 0.05
**Huoccup**					
Not-working(ref)					
Working	0.352	1.421	0.108	3.259	P ≤ 0.05
Wealth index					
Poor(ref)					
Medium	0.452	1.571	0.099	4.566	P ≤ 0.05
Rich	0.642	1.9	0.1	6.42	P ≤ 0.05
**Huedu**					
No education(ref)					
Primary	0.436	1.547	0.083	5.253	P ≤ 0.05
Secondary	0.645	1.906	0.154	4.188	P ≤ 0.05
Higher	0.178	1.195	0.182	0.978	P ≤ 0.05

*Statistically significant at 5%, ref=categorical reference

For instance, based on the result of the Bayesian multilevel random coefficient model in Table 2, Place of residence was identified as a prognostic factor for ANC service use of pregnant women. The odds ratio of pregnant women living in the rural areas being receiving ANC services is 0.206(OR = 0.206, P < 0.05). This shows that ANC usage for those pregnant women live in rural area were decreased by 79.4% than women's who were living in the urban areas. This means that pregnant women at reproductive age (15-49) who were living in rural areas were less likely to receive ANC services than those pregnant women living in urban areas. Similarly, the Odds ratio for Pregnant women with medium and rich wealth index is 1.571(OR = 1.571, P ≤ 0.05) and 1.90(OR = 1.90, P ≤ 0.05) respectively. This means that pregnant women who are with medium and rich wealth index levels had 57.1% and 90% increased odds compared to those pregnant women with poor index wealth consecutively. This implies that pregnant women who belong to the medium and rich wealth index category were more likely to participate in the usage of ANC service, when compared to those women of the poor wealth index. And similar interpretation can be given for the rest factors.

According to the results of fixed and random effect of Bayesian multilevel random coeffcient model showed in table 3 marital status, Comfortability of road, Use of mass media and Who advise you to use service have significant determinant of ANC service use of women. Based on the results showed in table 3, the estimated variance is the assessed difference of intercept, incline of marital status, comfortability of road, Use of mass media and Who advise you to use service are kebele wise captures and the slants fluctuate significantly. That is, there is a significant variety in the impacts of these logical factors across the areas (kebeles). A portion of the changes of the collaboration (interaction) terms among intercepts and inclines of independent factors are additionally found significant. Understanding of significant covariance terms can be handily made as far as the correlation coefficients showed in Table 3 and the correlation matrix contains the assessed connection between random intercepts and slants that implies contrarily corresponded. The negative sign for the relationship amongst captures (intercept) and frailty suggest that Kebele with higher captures (intercept) in general have on normal lower inclines on the comparing factors.

**Table 3:** Results for fixed and random effects of Bayesian multilevel random coefficient model

Covariates	Category	Exp($\hat{\beta }$)	S. E	Z-value	P-value	[95% Conf. Interval]	
**Marital status**	Married	1.8445	0.5548	3.32	P ≤ 0.05	0.757	2.932
	Divorced	1.1982	0.6144	1.95	P ≤ 0.05	-0.006	2.402
	Widowed	2.508	0.686	3.66	P ≤ 0.05	1.163	3.853
	Single (Ref)						
**Comfortability of road**	No	-1.718	0.3918	-4.39	P ≤ 0.05	-2.486	-0.9502
	Yes (Ref)						
**Use of mass media**	No	-2.641	0.3878	-6.81	P ≤ 0.05	-3.4014	-1.881
	Yes (Ref)						
**Who advise you to use** **service**	Husband	0.0705	1.5338	0.05	P ≤ 0.05	-2.9356	3.07679
	friends	-3.174	0.8057	-3.94	P ≤ 0.05	-4.753	-1.595
	family	-2.142	0.8876	-2.41	P ≤ 0.05	-3.882	-0.4028
	mass media	-4.719	0.965	-4.89	P ≤ 0.05	-6.611	-2.827
	from no one	-8.381	1.0249	-8.18	P ≤ 0.05	-10.39	-6.373
	Health professional						
**Intercept**		2.357	0.184	8.33	P ≤ 0.05	5.625	9.089
**Random part of estimate: Level-2 variance** **covariance**		Estimated variance		S. E	[95% Conf. Interval]		
							
$Var(u_{0j})=\sigma_{u0j}^{2}$		0.107		0.043	0.062		13.294
**var (*u*_11*j*_)**		0.173		0.223	0.721		6.548
**Cov (*u*_0*j*_, *u*_11*j*_)**		0.398		0.135			

## Discussion

In this study, the Authors applied both descriptive and Bayesian multilevel logistic regression random coefficient Models. Two-level models are considered, the first level was individuals (women) nested in higher levels, kebeles of the respondent. Doing this, the Authors tried to Survey the effect of kebeles variation on the usage of Antenatal care services.

The wealth index has had a significant effect on antenatal care service use of regenerative age women. Similar to the previous study conducted by 13 this study shows that women with poor wealth index levels are less accessible to the service. This shows that the women are discouraged with expenses regarding transportation and other health services like that of medication in order to utilize the service.

With respect to the education levels of the husband, pregnant women whose husband attains primary, secondary, and higher levels are more effective in using ANC service relative to no educated. This indicates that increasing husbands' education level is, in turn, reducing the chances of the women not using delivered services. This finding in line with the previous study conducted by 11 and found mothers having primary or higher education level husband have more chance to use ANC services than those mothers who had husband with no education. Because education plays a great role in enhancing women's as well as husbands' attitudes toward the use of modern health care services.

Husband's occupation was one of the most important determinants of Antenatal care service utilize among women in Bale zone Sinana district. According to findings, as compared with women whose Husband was not working, the risk of being ANC service use for women whose husband working was highly significant. This finding is consistent with other studies 15 showing that women of husbands in working level were more likely to receiving antenatal service.

Similarly, birth order has negatively related with ANC service use of pregnant women at reproductive age, 15-49. This indicates that women who have more number of children are less likely to seek the service compared to women who have 1-4 children. This result coincides with findings by 9 in Haramaya district, eastern Ethiopia.

## Conclusion

In this study, the authors applied the Bayesian Multilevel binary logistic regression model of the random coefficient to identify influential variables/factors which might influence the response variable, antenatal care service use of pregnant women. The study identified factors that determine the use of antenatal care services of pregnant women at regenerative age. The result showed that residence, wife beat, age, birth order, religion, husband occupation, wealth index, husband education, marital status, comfortability of road, use of mass media, and advice before start the service as statistically significant factors. Based on the finding of this study, there was a high variation among pregnant women not using antenatal care services and the probability of antenatal cares service use was found to rise with increasing husband occupation, wealth index, age category 25-34, and husband education level.
